# Effectiveness of a Phage Cocktail as a Potential Biocontrol Agent against Saprophytic Bacteria in Ready-To-Eat Plant-Based Food

**DOI:** 10.3390/v15010172

**Published:** 2023-01-06

**Authors:** Michał Wójcicki, Olga Świder, Iwona Gientka, Stanisław Błażejak, Paulina Średnicka, Dziyana Shymialevich, Hanna Cieślak, Artur Wardaszka, Paulina Emanowicz, Barbara Sokołowska, Edyta Juszczuk-Kubiak

**Affiliations:** 1 Laboratory of Biotechnology and Molecular Engineering, Department of Microbiology, Prof. Waclaw Dabrowski Institute of Agricultural and Food Biotechnology—State Research Institute, Rakowiecka 36 Street, 02-532 Warsaw, Poland; 2 Department of Food Safety and Chemical Analysis, Prof. Waclaw Dabrowski Institute of Agricultural and Food Biotechnology—State Research Institute, Rakowiecka 36 Street, 02-532 Warsaw, Poland; 3 Department of Biotechnology and Food Microbiology, Institute of Food Sciences, Warsaw University of Life Sciences (WULS-SGGW), Nowoursynowska 166 Street, 02-776 Warsaw, Poland; 4 Culture Collection of Industrial Microorganisms—Microbiological Resources Center, Department of Microbiology, Prof. Waclaw Dabrowski Institute of Agricultural and Food Biotechnology—State Research Institute, Rakowiecka 36 Street, 02-532 Warsaw, Poland; 5 Department of Microbiology, Prof. Waclaw Dabrowski Institute of Agricultural and Food Biotechnology—State Research Institute, Rakowiecka 36 Street, 02-532 Warsaw, Poland

**Keywords:** bacteriophages, biocontrol, saprophytic bacteria, food quality, minimally processed food, phage cocktail, spraying, absorption pad

## Abstract

This study aimed to evaluate the effectiveness of the phage cocktail to improve the microbiological quality of five different mixed-leaf salads: rucola, mixed-leaf salad with carrot, mixed-leaf salad with beetroot, washed and unwashed spinach, during storage in refrigerated conditions. *Enterobacterales* rods constituted a significant group of bacteria in the tested products. Selected bacteria were tested for antibiotic resistance profiles and then used to search for specific bacteriophages. Forty-three phages targeting bacteria dominant in mixed-leaf salads were isolated from sewage. Their titer was determined, and lytic activity was assessed using the Bioscreen C Pro automated growth analyzer. Two methods of phage cocktail application including spraying, and an absorption pad were effective for rucola, mixed leaf salad with carrot, and mixed leaf salad with beetroot. The maximum reduction level after 48 h of incubation reached 99.9% compared to the control sample. In washed and unwashed spinach, attempts to reduce the number of microorganisms did not bring the desired effect. The decrease in bacteria count in the lettuce mixes depended on the composition of the autochthonous saprophytic bacteria species. Both phage cocktail application methods effectively improved the microbiological quality of minimally processed products. Whole-spectral phage cocktail application may constitute an alternative food microbiological quality improvement method without affecting food properties.

## 1. Introduction

The improvement of consumers’ eating habits and the search for new health-promoting products have caused an increased interest in minimally processed food (MPF) [1,2,3]. These products are preserved using non-thermal techniques, which leads to limited changes in the food texture [4,5]. This approach also allows the preservation of bioactive food ingredients, such as vitamins, provitamins, and phytonutrients, that naturally occur in large amounts in plant-based minimally processed food products [6,7,8,9].

Minimal processing technology (MPT) uses several physical food preservation methods that include high hydrostatic pressure (HHP), radiation, ultrasonication (US) and pulsed electric field (PEF). *Sous-vide* and *cook-chill* packaging techniques, protective edible coatings, modified atmosphere packaging (MAP) and storage are also employed [10,11,12]. The use of the above methods of food preservation may negatively affect either the sensory characteristics or the nutritional value of the products. Moreover, not all methods effectively reduce the microbial contamination of preserved products. HHP has a bactericidal effect on vegetative cells, but spores are not completely inactivated without the use of additional techniques (e.g., heat treatment), which may be important in the case of contamination of juices with bacteria of the genus *Alicyclobacillus* [13,14,15,16] or other food products with bacteria of the genus *Bacillus* [17,18]. Ionizing radiation effectively reduces the growth of pathogenic bacteria, but even small doses (0.5 kGy) reduce the quality of leafy vegetables. Although the Food and Drug Administration (FDA) recommends high doses of radiation to effectively preserve iceberg lettuce, it can lead to cell sap leakage [19]. US also cannot be used to preserve minimally processed food of plant origin, because, like high doses of ionizing radiation, it causes leakage of cell contents into the environment. The release of cell juices into the packaging is a good medium for the growth and development of saprophytic microorganisms, naturally present on the surface of the products [20,21]. Intensification of saprophytic bacteria’s growth reduces the products’ quality (change of taste, smell, color) [20,21,22]. PEF enables the inactivation of microorganisms and simultaneously protects the desired sensory and physical characteristics of food products. However, PEF induces conformational changes in proteins and thus may affect the activity of native enzymes [23,24,25]. The quality of plant-derived products depends on the storage atmosphere [26]. Research confirms the positive impact of packaging in a controlled atmosphere (CA) on the sensory quality of food, especially in the context of red spots and the browning of leaf edges [27,28]. To sum up, the use of physical methods in the food industry does not always guarantee a food product with satisfactory sensory properties and microbiological parameters.

Biological methods that can be an alternative to physicochemical methods are gaining more and more attention regarding the preservation of minimally processed food with the highest microbiological risk, such as freshly squeezed juices, sprouts or salad mixes [3,21,29,30,31]. The concept of using protective microorganisms is already well known [3,30,31], but another approach could be the use of natural “enemies”, i.e., bacteriophages [21,29]. The use of bacteriophages can meet consumer expectations for minimally processed food by extending its shelf life without affecting its physical properties [20,22,29]. Bacteriophages are highly specific bacterial viruses and usually can infect only one species or strain of bacteria and, unlike antibiotics, do not destroy the natural commensal microbiota of the human digestive tract [21,29]. Research shows that phages are insensitive to many stress conditions during food processing (stability over a wide range of temperatures and pH) [20,21,29,32]. Their pH stability increases at lower temperatures [22]. Phages are used in three sectors of the food industry: primary production (mainly used to prevent the formation of biofilms on the surface of equipment), bio-sanitation (mainly used in production plants) and bio-preservation (used to extend the shelf life of products by combating pathogenic bacteria that spoil food) [21,22].

The success of using phages in food depends on overcoming several barriers. The number of phages represents an important factor that contributes to the efficacy of the application. Generally, the higher the phage concentration, the more significant the reduction of target bacteria [33]. Another obstacle is the food matrix used, which can affect phage performance. Liquid matrices allow better phage diffusion; however, the properties of the matrix remain pivotal. In the study by Zinno et al. [34], application of phages to liquid eggs resulted in reduced activity and decreased phage titer, which was explained by the high viscousness of the matrix that limited diffusion and uniform distribution of phage particles. The choice of the method of phages application may also be challenging. Phage cocktails were used in the form of dipping and spraying the products. Phages were dosed into food in the form of microcapsules, which protected them from hostile environmental factors, such as extreme pH values and temperature [33]. There are many challenges associated with the use of phages, but they can be overcome through the careful selection of phages included in biopreparations. One of the problems is the emergence of phage-resistant bacteria; therefore, manufacturing cocktails composed of several different phages with the broadest possible spectrum of antibacterial activity seems to be a relevant approach [29]. Phages can carry virulence genes or antibiotic resistance genes, but these problems can be avoided by using strictly lytic phages. There are also concerns about phage immunogenicity and cytotoxicity that may result from the lysis of the target bacteria [33].

Preparations based on bacteriophages are still not approved in the European Union for use in direct contact with food [35,36,37]. Many non-EU countries, including the USA, Brazil, the Netherlands, Israel, Canada, Switzerland, Australia, and New Zealand, have allowed the use of phages in the food industry, and numerous companies offer commercial phage biopreparations dedicated to the food industry [37,38]. Many preparations have been approved by the FDA, such as PhageGuard (e.g., Listex^TM^ P100, Secure Shield E1, EcoShield^TM^, ListShield^TM^, ShigaShield^TM^, SalmoFresh^TM^) and United States Department of Agriculture (USDA; PhageGuard, Finalyse^®^) [38,39,40]. Ten commercial phage preparations were given provisional GRAS status [41]. In 2016, EFSA released a report, in which safety and effectiveness in combating *Listeria monocytogenes* for Listex^TM^ P100 by Micreos Food Safety were confirmed [42], based on scientific research results [43,44,45,46].

It should be emphasized that commercial bacteriophage preparations are mainly targeted at pathogenic bacteria that do not predominate in the food environment. In the case of minimally processed food, a significant problem is a decrease in product quality due to the development of the accompanying saprophytic microbiota, which causes, among others, spoilage of products through rotting, deterioration of color and taste [20,21,22]. Saprophytic bacteria found in food can also be a reservoir of antibiotic resistance, which can be passed on to subsequent generations of bacteria, including those constituting the microbiota of the digestive tract of consumers [47,48,49,50].

Therefore, the main goal of the study was to evaluate the effectiveness of the phage cocktail to improve the microbiological quality of five different mixed-leaf salads, rucola, mixed-leaf salad with carrot, mixed-leaf salad with beetroot, washed and unwashed spinach, during refrigerated storage. First, we assessed the microbiological quality of selected minimally processed plant products in general, and then antibiotic resistance profiles for the dominant bacteria in the food products were determined. Finally, newly isolated specific bacteriophages were used to prepare a phage cocktail, and their effectiveness to improve the microbiological quality of the ready-to-eat (RTE) plant-based food products was assessed.

## 2. Materials and Methods

### 2.1. Research Material

The research material was commercially available lettuces and mixes with a minimum degree of processing. The research covered five products from a leading producer in the Polish market. In the case of four RTE products (rucola (RUC), mixed leaf salad with carrot (MLSC), mixed leaf salad with beetroot (MLSB) and spinach (washed spinach, WSP)), the manufacturer declared that the raw material was washed before it was hermetically packed. The fifth product was ready-to-eat spinach (unwashed spinach, UWSP) after washing by the consumer. The tested products were stored in refrigerated conditions recommended by the manufacturer (at 4–5 °C).

### 2.2. Evaluation of the Microbiological Quality of the Tested Minimally Processed Plant-Based Food Products

Assessment of the microbiological quality of the tested products was determined during refrigerated storage. All analyses were carried out in biological triplicate, on the expiry date (five days before the expiration date) and 48 h after the expiration date. The microbiological analysis included the determination of the total number of psychrophilic and/or psychrotrophic microorganisms (PCA, BTL, Poland), the total number of mesophilic microorganisms (PCA, BTL, Poland), the number of acidifying bacteria (Lactose and Chinese Blue Agar, BTL, Poland), the number of bacteria from the *coli* group (Endo Agar, BTL, Poland) and the number of bacteria from the *Enterobacteriaceae* family (VRBG Agar, BTL, Poland). In addition, the products were tested for the presence of pathogenic *Escherichia coli* O157:H7 (Fluorocult^®^ *E. coli* O157:H7-Agar, Merck, Germany), coagulase-positive staphylococci (according to PN-EN ISO 6888–1:2001 [51]), *Salmonella* sp. (according to PN-EN ISO 6579-1:2017 [52]), *Listeria monocytogenes* (according to PN-EN ISO 11290-1:2017 [53]) and *Cronobacter sakazakii* (according to ISO 22964:2017 [54]). Colonies grown on the media were counted and expressed as log CFU g^−1^.

### 2.3. Taxonomic Identification of the Isolated Bacterial Strains

After assessing the microbiological quality of the minimally processed products, bacterial colonies were selected for the isolation of pure bacterial cultures. Due to the high contamination with bacteria of the *Enterobacteriaceae* family, several dozen strains were isolated from the VRBG Agar and Endo Agar. Single colonies were repeatedly picked and streaked on nutrient agar (PCA, BTL, Poland), and cultured at 37 °C to ensure pure bacterial cultures, which were then identified. All strains were maintained at −80 °C in Luria Bertani broth (composition: 10 g L^−1^ of bactotryptone, 10 g L^−1^ of sodium chloride, and 5 g L^−1^ of yeast extract; pH 7.2 ± 0.2) supplemented with 20% glycerol. This article presents the identification of 43 bacterial host strains that were later used in this study. The strains were deposited in the Culture Collection of Industrial Microorganisms—Microbiological Resources Center (IAFB). Species membership was determined by amplification of the *16S* rRNA gene region. Bacterial DNA was isolated using a commercial DNeasy PowerFood Microbial Kit (Qiagen, GmbH, Hilden, Germany), and amplified with 16S–F (5′–AGAGTTTGATCCTGGCTCAG–3′) and 16S–R (5′–ACGGCTACCTTGTTACGACT–3′) primers [55]. The PCR conditions for the gene amplification were as follows: 2 min of initial denaturation at 95 °C, followed by 35 amplification cycles of denaturation at 94 °C for 30 s, hybridization at 51 °C for 35 s, and extension step at 72 °C for 1 min, ending with a final extension period of 72 °C for 10 min (SimpliAmp™ Thermal Cycler, Applied Biosystems™, ThermoFisher Scientific, Waltham, MA, USA). The amplicons were separated by electrophoresis on 1.5% agarose gel containing the SimplySafe™ interfering compound (5μL/100 mL; EURx, Gdansk, Poland). To estimate the size of the amplicons, 5 μL of a DNA Ladder in the range of 100–3000 bp was used (A&A Biotechnology, Gdansk, Poland). Electrophoresis was carried out at 80 V for 60 min using the Sub–Cell GT Horizontal Electrophoresis System (Bio–Rad, Madrid, Spain). The bands were visualized using the GeneFlash Network Bio Imaging System (Syngene, Wales, UK). Sequencing was outsourced to Genomed S.A. company (Poland). Raw sequences were analyzed using BLASTn (NCBI) and deposited in the GenBank database. Moreover, taxonomic identification of bacterial strains was performed using proteomic profiles generated by MALDI–TOF–MS (Matrix-Assisted Laser Desorption Ionization Time-of-Flight Mass Spectrometry) analysis (Shimadzu Biotech, Manchester, UK).

### 2.4. Antimicrobial Sensitivity Testing

Bacterial strains were tested in vitro for their susceptibility to 28 antimicrobial agents (Oxoid, Hampshire, UK). Antimicrobial susceptibility tests were performed using a Kirby–Bauer disk diffusion method according to the European Committee on Antimicrobial Susceptibility Testing (EUCAST) [56] and Clinical and Laboratory Standards Institute (CLSI) [57] standards on Mueller–Hinton agar (Merck). The plates were incubated at 37 °C for 18 ± 2 h. The following antimicrobial agents belonging to eight different classes were tested: (1) penicillins: ampicillin (AMP, 10 μg), sulbactam/ampicillin (SAM, 20 μg), amoxicillin/clavulanic acid (AMC, 30 μg), piperacillin (PRL, 30 μg), piperacillin/tazobactam (TZP, 36 μg), ticarcillin/clavulanic acid (TTC, 85 μg); (2) cephalosporins: cefepime (FEP, 30 μg), cefotaxime (CTX, 5 μg), ceftaroline (CPT, 5 μg), ceftazidime (CAZ, 10 μg), ceftazidime/avibactam (CZA, 14 μg), ceftolozane/tazobactam (CT, 40 μg), ceftriaxone (CRO, 30 μg); (3) carbapenems: ertapenem (ETP, 10 μg), imipenem (IPM, 10 μg), meropenem (MEM, 10 μg); (4) monobactams: aztreonam (ATM, 30 μg); (5) fluoroquinolones: ciprofloxacin (CIP, 5 μg), pefloxacin (PEF, 5 μg), levofloxacin (LEV, 5 μg), moxifloxacin (MXF, 5 μg), ofloxacin (OFX, 5 μg), norfloxacin (NOR, 10 μg); (6) aminoglycosides: amikacin (AK, 30 μg), gentamycin (CN, 10 μg), tobramycin (TOB, 10 μg); (7) phenicols: chloramphenicol (C, 30 μg); and (8) sulfonamides: sulphamethoxazole/trimethoprim (SXT, 25 μg). The tests were made in triplicate and the mean diameter of the inhibitory zones was calculated. Susceptibility of the isolates to antimicrobial agents was categorized (as susceptible or resistant) by measurement of the inhibition zone, according to interpretive criteria that adhered to the EUCAST guidelines. *Escherichia coli* ATCC 25922 was used as the reference strain. Bacterial strains resistant to three or more different antimicrobial classes were categorized as multidrug-resistant (MDR) isolates.

Multiple antibiotic resistance (MAR) phenotypes were recorded for bacterial strains showing resistance to more than two antibiotics, and the MAR index [58] was calculated as:

(1) $$\mathrm{MAR}=\frac{\mathrm{Number}\;\mathrm{of}\;\mathrm{resistance}\;\mathrm{to}\;\mathrm{antibiotics}}{\mathrm{Total}\;\mathrm{number}\;\mathrm{of}\;\mathrm{antibiotics}\;\mathrm{tested}}$$

### 2.5. Screening for Phenotypic Detection of β–Lactamases-Producing Bacterial Strains

The phenotypic assessment of the ability to produce β–lactamases by bacterial strains was carried out. Phenotypic detection of ESBL-producing (extended-spectrum beta-lactamases) strains was performed by the double-disc synergy test (DDST) on Mueller–Hinton agar (Merck) with amoxicillin/clavulanic acid (AMC, 30 μg), cefepime (FEP, 30 μg), cefotaxime (CTX, 30 μg), and ceftazidime (CAZ, 30 μg) disks (Oxoid, Hampshire, UK). Samples were classified as ESBL-positive when the inhibition zone around cefotaxime or ceftazidime increased toward the central disk with AMC [59]. Moreover, for the detection of ESBL- and carbapenemases-producing strains, commercial selective media were used: CHROMagar ESBL and CHROMagar mSuperCARBA, respectively (Graso Biotech, Starogard Gdanski, Poland). *Escherichia coli* ATCC 25922 was used as the negative control.

### 2.6. Bacteriophage Isolation, Purification, and Propagation

The phages have been isolated from municipal sewage, which was collected from the wastewater treatment plant in Izabelin “Mokre Łąki” (near Warsaw, Poland). A total of 25 mL of municipal sewage was centrifuged at 10,000× *g* (20 °C for 10 min) to separate organic and mineral particles from bacteria and potential bacteriophages. The supernatant was filtered using a syringe filter with a membrane pore diameter of 0.22 μm (Sartorius, Germany). Then, 20 mL of the filtrated supernatant containing bacteriophages from the sewage were transferred to 20 mL of double-concentrated T-broth (composition: 8 g L^−1^ of enriched broth, 5 g L^−1^ of peptone, 5 g L^−1^ of sodium chloride, and 1 g L^−1^ of glucose). The culture medium with bacteriophages was inoculated with 1 mL of an overnight culture of bacterial strain on Luria Bertani broth and incubated at 37 °C for 24 h. Afterwards, the culture was centrifuged at 8000× *g* for 10 min to separate bacteria from the proliferated bacteriophages. The supernatant was filtered using a syringe filter with a membrane pore diameter of 0.45 μm (Sartorius, Germany; according to Mirzaei and Nilsson procedure [60] with modification) and freeze-stored (–80 °C) with 20% addition of glycerol.

### 2.7. Evaluation of the Lytic Activity of Phages against Bacterial Hosts

The lytic activity of bacteriophages against the isolated bacterial strains was determined with a double-layered plate according to the method of Jamal et al. [61]. Glass tubes containing dissolved LCA (composition: 10 g L^−1^ of bactotryptone, 10 g L^−1^ of sodium chloride, 5 g L^−1^ of yeast extract, and 7 g L^−1^ of agar-agar) were placed in a water bath at 48 °C until the temperature was equilibrated. Then, 100 μL of 0.025 M CaCl_2_ and 0.025 M MgSO_4_ were added to sterile test tubes and incubated with 100 μL of overnight bacterial culture. Then, 500 μL of diluted phage lysate was transferred to test tubes. After 20 min, the tubes were supplemented with 4 mL of soft LCA agar (48–52 °C), mixed, and then poured onto the first nutrient agar layer and incubated at 37 °C for 24 h. The unit used to measure phage titer was the plaque-forming unit per mL (PFU mL^−1^). Single-phage (clear with or without halo zone or turbid) plaques were cut with a scalpel and purified in SM buffer according to the method proposed by Mirzaei and Nilsson [60]. Purification was performed in four rounds of single-plaque passage to ensure that the isolate represented the clonal phage population. In addition, after filtering through a syringe filter with a membrane pore diameter of 0.45 μm, each lysate was inoculated to check for possible contamination with bacterial cells. Growth curves were made for each of the bacterial strains (data unpublish in this study). For this purpose, the harvested culture was inoculated on PCA medium every hour, incubated at 37 °C for 24 h, and the optical density was measured simultaneously. The dependence of the optical density on the number of bacterial cells was determined (performed in triplicate). Next, coefficients of the specific growth rate (μ) were computed for each strain using the following formula:

μ = (ln OD_max_ − ln OD_min_)/t, (2)

where ln OD_max_—natural logarithm of the maximal value of the optical density of the culture during the exponential growth phase; ln OD_min_—natural logarithm of the minimal value of the optical density of the culture during the exponential growth phase; t—duration of the exponential growth phase, (h).

Once phage titer was determined and bacterial host growth curves were plotted, bacteria growth kinetics was measured using a Bioscreen C automated growth analyzer (Yo AB Ltd., Growth Curves, Helsinki, Finlandia). Bacteria proliferated in the Luria Bertani broth. The culture was diluted at a ratio of 1:100 in a fresh culture medium with the addition of CaCl_2_ and MgSO_4_, both having final concentrations of 0.01 M. To ensure the optimal value of the multiplicity of infection (MOI) coefficient, flasks with the new culture were incubated at a temperature of 37 °C with continuous shaking until the desired optical density depended on the phage titer. Then, 180 μL of each culture was pipetted into multi-well plates and incubated in a Bioscreen C at 37 °C until optical density increased by OD_600_ ~0.1 compared to the control medium. Phage lysates were prepared so that the value of the MOI coefficient reached 1.0 and 0.1, respectively. Then, 20 μL of respective phage lysates were added to wells, left at 20 °C for 15 min to allow the phages to adsorb to the host cell surface, and incubated at 37 °C for 24 h. The apparatus measured optical density automatically every 15 min, at a wide band of wavelengths ranging from 420 to 580 nm, with 15 s shaking preceding each readout. The test was performed in 10 replicates for each strain and infection rate.

### 2.8. Application of the Phage Cocktail to the Analyzed RTE Food Products

The entire study was based on the proprietary methodology. The phage cocktail was a mixture of 43 isolated bacteriophages with known titer lysates. Before infecting the products, the phage mixture was filtered through a cellulose syringe filter with a membrane pore diameter of 0.45 μm. The titer of the prepared phage cocktail equaled 2.1 × 10^8^ PFU mL^−1^ and that was the average number of all phages in the prepared mixture. Products were infected in two ways. The first way, 50 g of the product was applied to the styrofoam tray and a direct spray (5 mL) of phage cocktail was carried out. The samples prepared were packed in a protective atmosphere. In the second case, a cellulose absorption pad with a capacity of 2500 mL m^−2^ (5 cm × 4 cm; 5 mL of phage cocktail) impregnated with a phage suspension was placed on a styrofoam tray. Then, 50 g of the product was applied and packed in a protective atmosphere. All samples were stored at 20 °C. Measurements of changes in the total number of bacteria (TNB) were carried out in triplicate at the 0th, 6th, 24th, and 48th hours of the experiment on nutrient agar. Simultaneously, the control samples (without the addition of the phage cocktail) were performed.

### 2.9. Statistical Analyses 

Obtained data were statistically assessed using Statistica 13.3 software suite (TIBCO Software Inc., Palo Alto, CA, USA). Analysis of variance (ANOVA) followed by Tukey’s test post hoc (*p* < 0.05) was performed independently for each microbiological analysis and each RTE food product to define homogenous groups, which in the table have been marked with identical letters. In the analysis of the practical application of the phage cocktail, a two-way analysis of variance with a confidence interval of 0.95 was performed.

## 3. Results and Discussion

### 3.1. Evaluation of the Microbiological Quality of the Tested Minimally Processed Plant-Based Food Products

This study aimed to assess the microbiological quality of minimally processed plant-based food products and their variability during refrigerated storage. Changes in the number of specific groups of microorganisms determined within the best-before date and 2 days after its end are summarized in Table 1. 

All tested RTE food products were characterized by a high number of psychrophilic and/or psychrotrophic saprophytic microorganisms. The microbes showed active metabolism and actively multiplied. Generally, two days after the expiration date, the level of contamination increased by an average of 1–2 log units compared to the samples analyzed within the expiration date. Among the analyzed products, rucola was the least microbiologically stable. Its contamination level with psychrophilic and/or psychrotrophic microorganisms increased by 2 log units, from 7.14 ± 0.37 log CFU g^−1^ (BED) to 9.45 ± 0.45 log CFU g^−1^ (AED). During the shelf-life (BED), the lowest level of psychrophilic and/or psychrotrophic microorganisms was found in unwashed spinach (6.29 ± 0.05 log CFU g^−1^), and the highest in washed spinach (7.61 ± 0.52 log CFU g^−1^). The number of mesophilic microorganisms, as in the case of psychrophiles and/or psychrotrophs, increased by an average of 1–2 log units during refrigerated storage (AED). The least microbiologically stable was rucola, the contamination of which increased by 2 log units, from 7.59 ± 0.59 log CFU g^−1^ (BED) to 9.84 ± 0.88 log CFU g^−1^ (AED). Unwashed spinach within the shelf life (BED) was characterized by the highest level of contamination with mesophilic microorganisms. As the only tested product, it was not intended for direct consumption by the consumer (due to the omission of the washing stage during the production process). During refrigerated storage, mesophilic levels increased from 10.10 ± 0.04 log CFU g^−1^ (BED) to 10.41 ± 0.06 log CFU g^−1^ (AED). A high level of microbial contamination indicates insufficient cleaning of products during the production process. The level of contamination is directly related to the initial microbial composition of the raw materials, which varies from batch to batch. Polish legislation does not specify the acceptable level of contamination with saprophytic microorganisms in plant-origin products with a minimum degree of processing. Taking into account the standards of other EU countries (e.g., France or Germany), where the permissible level is 5.0 × 10^7^ CFU mL^−1^, it should be noted that all of the tested products (within their shelf life) met this requirement in terms of psychrophilic microorganisms and/or psychrotrophic and mesophilic, except washed spinach. After the expiry date, none of the analyzed products met this criterion. In the study by Michalczyk and Macura [62], concerning the microbiological quality of leafy vegetables, it was found that the recommended German and French criteria for the total number of psychrophilic and/or psychrotrophic microorganisms were exceeded. Rucola already on the first day of the analysis contained 10^8^ CFU g^−1^ of these microorganisms, and their number slightly increased during refrigerated storage. Regarding iceberg lettuce, the level of psychrophilic and/or psychrotrophic microorganisms increased from 10^5^ CFU g^−1^ to 10^8^ CFU g^−1^. In the study by Maffei et al. [63], the contamination of leafy vegetables with mesophilic microorganisms was comparable to the shelf-life products tested in our study and ranged from 10^6^ CFU g^−1^ to 10^7^ CFU g^−1^. The level of contamination with mesophilic microorganisms in the study by Abadias et al. [64] in washed spinach and lettuce was also comparable to the results obtained in our study and amounted to 10^7^ and 10^6^ CFU g^−1^, respectively.

Another group of microorganisms studied were acidifying bacteria, the number of which increased in each product during refrigerated storage, by an average of 1 to 4 log units. No significant difference was found in the number of acidifying bacteria before and after the expiration date in the mixed leaf salad with carrot and washed spinach. At the shelf life (BED), the highest level of acidifying bacteria was found in the mixed leaf salad with carrot (6.20 ± 1.21 log CFU g^−1^), and the lowest in the mixed leaf salad with beetroot (5.17 ± 0.89 log CFU g^−1^). During refrigerated storage, the level of acidifying bacteria increased the most in unwashed spinach, from 5.98 ± 0.15 log CFU g^−1^ (BED) to 9.84 ± 0.05 log CFU g^−1^ (AED). The unwashed spinach, compared to the washed spinach, was characterized by a higher level of acidifying bacteria. In the study of Abadias et al. [64], the level of contamination with acidifying bacteria was lower than specified in our study and amounted to 10^5^ CFU g^−1^ for spinach and 10^4^ CFU g^−1^ for lettuce.

During the shelf life (BED), the highest level of coliform bacteria was found in the mixed leaf salad with carrot (6.18 ± 0.57 log CFU g^−1^), and the lowest in unwashed spinach (4.20 ± 0.19 log CFU g^−1^). No significant difference was found in the number of acidifying bacteria before and after the expiration date in the mixed leaf salad with carrot and washed spinach. A high level of coliform bacteria may indicate fertilization of the agricultural field with natural fertilizers and their insufficient removal from the surface of the raw material during the production process. During refrigerated storage, the level of lactose-positive rods increased by an average of 1–2 log units.

In the course of the shelf life (BED), washed spinach and the mixed leaf salad with carrot contained the highest number of bacteria from the *Enterobacteriaceae* family (6.31 ± 0.08 log CFU g^−1^ and 6.26 ± 0.51 log CFU g^−1^, respectively). The lowest contamination was found in unwashed spinach (5.32 ± 0.09 log CFU g^−1^), in which, during refrigerated storage (AED), the level of *Enterobacteriaceae* increased to 5.98 ± 0.06 log CFU g^−1^. In the remaining products, during refrigerated storage, the number of bacteria from the *Enterobacteriaceae* family increased by 1–2 log units; however, no significant growth of this group of bacteria was observed during storage in washed spinach. In the study by Abadias et al. [64], the level of *Enterobacteriaceae* in rucola and spinach was 5.3 log CFU g^−1^ and 6.0 log CFU g^−1^, respectively. The much lower level of *Enterobacteriaceae* (4.4 log CFU g^−1^) was determined in lettuce. In the study of iceberg lettuce conducted by Michalczyk and Macura [62], contamination with bacteria belonging to the *Enterobacteriaceae* family was at the level of 10^4^ CFU g^−1^ to 10^6^ CFU g^−1^ during storage.

No pathogenic *Escherichia coli* O157:H7 was found in any of the tested food products. The products were free of coagulase-positive staphylococci in 1 g of food sample. *Salmonella* spp. and *Listeria monocytogenes* were not found in 25 g of food products. Other bacteria belonging to the genus *Listeria* spp. were isolated only from the mixed leaf salad with carrot. In each of the RTE food products, lactose-positive *Enterobacter* spp. or *Cronobacter sakazakii* were present.

### 3.2. Taxonomic Identification of the Isolated Bacterial Strains

Based on the evaluated microbiological quality of the products, it was decided to search for phages against dominant bacteria from the *Enterobacteriaceae* family. For this purpose, pure cultures of host bacterial strains were isolated, and the taxonomic affiliation of all strains was confirmed by either molecular methods (amplification of the *16S* rRNA gene region) or the analysis of proteomic profiles (using MALDI–TOF–MS). All nucleotide sequences of the strains have been deposited in the GenBank database (Table 2). Bacteria strains for which specific bacteriophages were isolated in the subsequent stages of this study are included in Table 2.

A total of 43 bacteriophages were found, of which six (*n* = 6), nine (*n* = 9), thirteen (*n* = 13), six (*n* = 6) and nine (*n* = 9) were specific to bacteria isolated from rucola, mixed leaf salad with carrot, mixed leaf salad with beetroot, washed spinach and unwashed spinach, respectively. Isolated bacteria belonged to seven genera from three families of *Enterobacterales* order: *Enterobacter* (*n* = 10), *Escherichia* (*n* = 10), *Citrobacter* (*n* = 2), and *Raoultella* (*n* = 1) from *Enterobacteriaceae* family; *Serratia* (*n* = 11), and *Rahnella* (*n* = 3) from *Yersiniaceae* family; and *Pantoea* (*n* = 6) from *Erwiniaceae* family. 

Genetic identification (*16S* rRNA amplification) of most bacteria strains coincided with proteomic identification. For three strains (i.e., *Citrobacter* sp. KKP 581, *Enterobacter* sp. KKP 3891, and *Enterobacter* sp. KKP 3892), the genetic identification allowed us to classify bacterial isolates only to the genus level. This may be related to the close phylogenetic relationship of strains belonging to the *Citrobacter* and *Enterobacter* genera. In addition, concerning the *Enterobacter ludwigii* KKP 3083 strain, proteomic identification based on MALTI-TOF-MS classified this strain as *Klebsiella oxytoca* (but only 84.4% of the match, which could have affected such an identification result). Similarly, for several isolates, MALDI-TOF-MS analysis classified the strains only to the genus level. This may be related to the product processing technology (e.g., during the treatment of food products, bacterial cells could be damaged, which could affect the identification result based on protein profiles). In addition, MALDI-TOF-MS analyzes protein spectra based on databases dedicated mainly to the medical industry, whereby medical isolates may be quite different from those isolated from food products.

### 3.3. Antimicrobial Sensitivity Testing and Screening for Phenotypic Detection of β–Lactamases-Producing Bacterial Strains

Antibiotics are usually used in the treatment of infections of bacterial etiology, and their widespread use in recent decades has led to a huge problem related to the antibiotic resistance of both pathogens and saprophytic bacteria [65,66,67,68]. In our study, bacteria strains were tested for susceptibility to twenty-eight antimicrobial agents belonging to eight different classes (Table 3). Among tested strains, seventeen (39.5%) showed no phenotype resistance to any of the tested antibiotics. All strains were sensitive to piperacillin, piperacillin/tazobactam, and ticarcillin/clavulanic acid (penicillins); cefotaxime, and ceftazidime/avibactam (cephalosporins); all tested carbapenems (i.e., ertapenem, imipenem, meropenem); aztreonam (monobactams); levofloxacin (fluoroquinolones); chloramphenicol (phenicols); and sulphamethoxazole/trimethoprim (sulfonamides).

In this study, most of the bacterial strains showed a MAR (Multiple Antibiotic Resistance) index lower than 0.3, whereas three strains showed a MAR index above 0.3: *Rahnella aquatilis* KKP 1383 isolated from mixed leaf salad with beetroot (MAR index = 0.36), *Serratia marcescens* KKP 585 isolated from mixed leaf salad with beetroot (MAR index = 0.32), and *Pantoea agglomerans* KKP 590 isolated from mixed leaf salad with carrot (MAR index = 0.32). Moreover, a low prevalence of MAR was observed among the strains; 18.6% (8/43) of the isolates were MDR (Multi-Drug Resistant). *Rahnella aquatilis* strain KKP 1383 exhibited the most extensive resistance profile to 10 antibiotics (AMP-CPT-CT-CRO-CIP-PEF-MXF-NOR-AK-TOB), belonging to 4 different classes of antibiotics (penicillins, cephalosporins, fluoroquinolones, and aminoglycosides). Extensive resistance profiles were also exhibited by *Serratia marcescens* strain KKP 585 and *Pantoea agglomerans* strain KKP 590, which were resistant to 9 antimicrobials (*Serratia marcescens* strain KKP 585: AMP-AMC-CPT-CT-PEF-MXF-AK-CN-TOB, in turn, *Pantoea agglomerans* strain KKP 590: AMP-AMC-FEP-CPT-CAZ-CT-PEF-MXF-AK) from 4 different classes of antibiotics (penicillins, cephalosporins, fluoroquinolones, and aminoglycosides). Some antibiotics were completely ineffective against tested bacteria (unpublished data). *Enterobacter cloacae* strains KKP 575, KKP 3684, KKP 3686, KKP 3692 and KKP 3706; *Enterobacter* sp. strain KKP 3892; and *Serratia marcescens* strains KKP 585, and KKP 671 showed full growth with ampicillin discs. Amoxicillin/clavulanic acid did not inhibit the growth of *Enterobacter cloacae* strain KKP 3692 and *Enterobacter* sp. strain KKP 3892. Moreover, *Serratia marcescens* strain KKP 585 was resistant to all tested antibiotics from the aminoglycosides class (i.e., amikacin, gentamycin, and tobramycin) (Table 3). Bacterial strains showed the highest resistance to selected antibiotics from the penicillin and cephalosporins classes (Table 4). Twenty (46.5%) strains exhibited resistance against ampicillin (penicillins), whereas fifteen (34.9%) were resistant to amoxicillin/clavulanic acid (penicillins) and ceftaroline (cephalosporins). Antibiotic resistance among pathogenic bacteria from the *Enterobacteriaceae* family isolated from food products has been studied by many researchers, but there are no reports on the resistance profiles of saprophytic bacteria isolated from minimally processed plant-based food. In the study by Richter et al. [69], the resistance profiles of bacteria from the *Enterobacteriaceae* family isolated from fresh vegetables were determined. Antibiotic resistance phenotypic analysis showed that 96.1% of the 77 selected isolates were MDR, while resistance to aminoglycosides (94.8%), chloramphenicol (85.7%) and tetracyclines (53.2%) was the most common. Vincenti et al. [70] determined antibiotic resistance in ready-to-eat food collected from hospital and community canteens in Rome. Studies have shown that approximately 38% of RTE food supplied in municipal canteens does not meet the microbiological criteria for food safety and may pose a particular threat to consumers due to the spread of antibiotic-resistant strains.

In our studies, no activation of ESBL-type antibiotic resistance mechanisms and carbapenemases-producing strains were observed. In a study by Richter et al. [69], positive phenotypic analysis of ESBL production was found in as many as 61 (79.2%) out of 77 tested isolates. In general, the strains isolated in our studies did not show high rates of multidrug resistance, but due to the risk of transmission of resistance to antibiotics in the environment, this situation should be monitored.

### 3.4. Evaluation of the Lytic Activity of Phages against Bacterial Hosts

Specific bacteriophages against the dominant saprophytic bacteria were sought. Each of the isolated bacterial strains was used as a potential host for the amplification of phage particles. Forty-three specific bacteriophages were isolated from municipal sewage. The presence of phages in the lysate filtered after multiplication was determined by spotting. A characteristic, large plaque in the spot where the lysate was initially instilled on the plate with the inoculated host bacterial strain indicated the presence of bacteriophages in the lysate. Spot-positive tests were confirmed by conventional plating of a series of lysate dilutions on nutrient agar plates. After incubation, the resulting plaques were counted and, considering the dilution, they were converted to the phage titer in the lysate. Table 5 presents bacterial host strains isolated from RTE food products along with the concentration of phages (i.e., phage titer) specific to them.

The lytic activity of isolated phages was also determined with the use of a Bioscreen C automated growth analyzer. For this purpose, growth curves were prepared for each strain (unpublished data), thanks to which it was possible to select the appropriate infection rate for each phage (MOI 1.0 or 0.1). Optical density measurement made with the use of the Bioscreen C automated growth analyzer allowed us to determine the start and duration of the logarithmic growth phase of the tested bacterial strains, which were intentionally infected with complementary phages with an infection rate of MOI of 1.0 and 0.1, respectively, compared to the control culture. Four of the phages studied (i.e., *Enterobacter* phage KKP 3262 against *Enterobacter cloacae* strain KKP 3082, *Enterobacter* phage KKP 3263 against *Enterobacter ludwigii* strain KKP 3083, *Serratia* phage KKP 3264 against *Serratia fonticola* strain KKP 3084, and *Citrobacter* phage KKP 3664 against *Citrobacter freundii* strain KKP 3655) were comprehensively characterized in our previous study [20], and genome sequences have been deposited in the GenBank database under the accession numbers OK210076, OK210074, OK210077, and OK210075, respectively. In addition, another three enzyme-producing phages (i.e., *Serratia* phage KKP 3708 against *Serratia liquefaciens* strain KKP 3654, *Serratia* phage KKP 3709 against *Serratia marcescens* strain KKP 3687, and *Enterobacter* phage KKP 3711 against *Enterobacter cloacae* strain KKP 3684) were described by Shymialevich et al. [71].

Table 5 shows changes in the optical density of cultures of strains isolated from selected RTE food products after the addition of phages. Lower coefficients of the specific growth rate in samples infected with phages indicate a significant reduction in cell division during the logarithmic growth phase of the tested strains. The use of an infection rate of MOI 1.0 was more potent in inhibiting bacterial host cell division compared to a lower infection rate (MOI 0.1). Culturing *Citrobacter freundii* strain KKP 3655 together with complementary phages with an MOI 1.0 reduced the specific growth rate almost two-fold compared to the control culture. Among the strains isolated from rucola, in the control culture (without the addition of phage), the *Citrobacter freundii* strain KKP 3655 started the logarithmic growth phase the fastest [20], and its duration was 10 h. During this time, the optical density increased by 0.255. The use of an infection factor of 1.0 most effectively delayed the start of the log-phase (after 12 h) [20], which lasted for 11 h 30 min, and the optical density of the culture increased by 0.136. 

Among the strains isolated from the mixed leaf salad with carrot, in the control culture (without the addition of phage), the *Enterobacter cloacae* strain KKP 3082 started the logarithmic growth phase the fastest (after 3 h 30 min), and its duration was 14 h 30 min [20]. During this time, the optical density increased by 0.312. The use of an infection factor of 1.0 most effectively delayed the start of the log-phase (after 7 h 45 min), which lasted for 14 h 45 min, and the optical density increased by 0.147. In the control culture (without the addition of phage), the *Enterobacter ludwigii* strain KKP 3083 began the logarithmic growth phase after 1 h 45 min, and its duration was 17 h 30 min [20]. During this time, the optical density increased by 0.486. The use of an infection factor of 0.1 most effectively delayed the start of the log-phase (after 6 h 15 min), which lasted for 13 h 15 min, and the optical density increased by 0.206. The logarithmic growth phase in samples with an infection rate of 1.0 started at 5 h 45 min and lasted 16 h 15 min. During this time, the optical density increased by 0.160. Regarding *Escherichia coli* strain KKP 3824, a lower coefficient of specific growth rate was observed at MOI 0.1 than at MOI 1.0 (0.008 and 0.009, respectively). This may be related to the fact that the phage titer was slightly higher, and the lytic activity of the phage might decrease.

Among the strains isolated from the mixed leaf salad with beetroot, in the case of infection with the phage of *Citrobacter* sp. strain KKP 581, there was an increase in the specific growth rate coefficient from 0.011 for the control culture to 0.015 for the sample at MOI 1.0, which may indicate the acquisition of bacterial resistance to this phage, lysogenization or pseudolysogeny phenomena. The presence of a too-high concentration of phage in the vicinity of the population of the strain could be a stress factor and cause the activation and increase in the rate of cell division. For an infection rate of MOI 0.1, phages reduced the number of cell divisions almost two times compared to the control culture. The use of an infection factor of 1.0 in most strains has a stronger inhibitory effect on bacterial host cell division compared to a lower infection factor (MOI 0.1). *Escherichia coli* strain KKP 3688, *Roaultella aquatilis* strain KKP 3689 and *Escherichia coli* strain KKP 3705 had a lower specific growth rate at MOI 0.1 than MOI 1.0, similar to *Escherichia coli* strain KKP 3824. Cultivation of *Serratia fonticola* strain KKP 3084 combined with complementary phages with an MOI of 1.0 reduced the specific growth rate factor almost five times, and at MOI 0.1 more than twice compared to the control culture. In the control culture (without the addition of phage), the *Serratia fonticola* strain KKP 3084 started the logarithmic growth phase after 5 h 30 min, and its duration was 14 h 30 min. During this time, the optical density increased by 0.464. The use of an infection factor MOI 1.0 accelerated the onset of the log-phase (after 1 h 15 min), which lasted for 21 h 15 min, and the optical density increased by 0.102. The phase of logarithmic growth in samples with MOI 0.1 started after 2 h 15 min and lasted 13 h 30 min. During this time, the optical density increased by 0.173.

Changes in the logarithmic growth phase using the Bioscreen C automated growth analyzer in phage-infected cultures have been extensively studied [22,72,73,74,75,76,77,78,79]. Zhao et al. [72] showed that regardless of the level of infection, bacterial cultures treated with phages started the logarithmic phase much later than control cultures. In a study conducted by Mahmoud et al. [73] growth of *Salmonella* Kentucky infected with bacteriophages at MOI 1.0 was delayed by all tested phages compared to control cultures. After a 24 h incubation, the phages completely inhibited the growth of the host bacterial strain. On the other hand, in an experiment conducted by Yu et al. [74], phage-infected cultures showed poorer growth compared to the control culture for up to 24 h. In the next 24 h, some of the cultures showed more robust growth than the control culture, which, according to the authors, resulted from acquiring bacteria resistance to infection with the tested phages. Xu et al. [77] used the Bioscreen C automated growth analyzer to evaluate the biphasic cocktail as a therapeutic strategy in *Edwardsiella* infections. In other studies [79], a Bioscreen C growth analyzer was used in an in vitro lysis assay of ValSw3-3 phage on *Vibrio alginolyticus* at different MOIs (10^−6^, 10^−3^, 10^−2^ and 10^−1^). The killing curve indicated that ValSw3-3 was highly effective against the host at all MOIs tested in this study and hardly any bacterial growth was observed for the first 12 h after infection. Generally, the repopulation of bacteria after phage infection implies the emergence of phage resistance, but during the experiment, regrowth of the bacterial population occurred quite slowly after infection.

### 3.5. Application of the Phage Cocktail to the Analyzed RTE Food Products

In the last stage of the research, the effectiveness of the phage cocktail (consisting of all 43 isolated bacterial viruses) was tested to reduce the number of bacteria in the tested food products (Table 6). It is worth mentioning that the phages used were isolated against bacterial hosts originating from lettuces of other production batches. Therefore, their action was possible only if the same bacterial strains were present in the product as in the production batches from which pure cultures were isolated, or if the phages showed a broad spectrum of activity against different species (or strains) of bacterial hosts.

During the 48-hour storage of rucola, the total number of bacteria in the control samples increased steadily from 8.47 ± 0.11 log CFU g^−1^ to 11.49 ± 0.13 log CFU g^−1^. The use of spraying and an absorption pad significantly reduced the growth of microorganisms in the product environment after 6 h. Both tested methods of application similarly limited the growth of bacteria. After 48 h, the TNB in the product was 9.46 ± 0.06 log CFU g^−1^ and 9.52 ± 0.08 log CFU g^−1^, respectively. During storage, TNB in the control samples increased by 3 log units, while the use of a phage cocktail in the form of direct spraying or absorption pad reduced the TNB by 99% compared to the control samples (reduction by 2 log units).

During the 48-hour storage of the mixed leaf salad with carrot, in the control samples, the TNB increased by almost 3 log units, from 8.00 ± 0.03 log CFU g^−1^ to 11.71 ± 0.06 log CFU g^−1^. The use of spraying and an absorption pad did not result in a significant limitation of the bacteria growth after 6 h, while after 48 h there was a significant reduction in the growth of TNB in the product environment. The absorption pad reduced the growth of bacteria in the food product more efficiently than spraying (the TNB after 48 h was 8.67 ± 0.14 log CFU g^−1^ and 9.07 ± 0.34 log CFU g^−1^, respectively). During storage, TNB in the phage spraying assays increased by one log unit. The use of an absorption pad during storage almost completely reduced the growth of bacteria (*p* < 0.05). The use of a phage cocktail in the form of direct spraying or an absorption pad reduced the TNB by 99.9% compared to control samples.

During the storage of the mixed leaf salad with beetroot, the TNB increased from 8.49 ± 0.13 log CFU g^−1^ to 11.78 ± 0.01 log CFU g^−1^ (more than 2 log units). Application of spraying and absorption pad had an insignificant effect limiting the growth of TNB after 6 h, while after 48 h there was a significant (*p* < 0.05) reduction in the growth of bacteria in the product environment. Spraying reduced the growth of bacteria in the product more effectively than the absorption pad (after 48 h TNB was 9.33 ± 0.56 log CFU g^−1^ and 9.44 ± 0.07 log CFU g^−1^, respectively). The use of a phage cocktail in the form of direct spraying or an absorption pad reduced the TNB by 99% compared to the control samples (reduction by 2 log units).

During the 48-hour storage of washed spinach, the TNB in control samples increased from 7.73 ± 0.14 log CFU g^−1^ to 8.63 ± 0.16 log CFU g^−1^. Application of spraying and absorption pad after 6 h significantly reduced the growth of bacteria, while after 24 h and 48 h the limiting effect was not achieved. Likely, the high water activity that was generated during the introduction of the spraying and soaked pad resulted in the growth of other bacteria associated with the product against which the phages were not specified.

Application of spraying and absorption pad to unwashed spinach significantly limited the growth of TNB after 6 h and 24 h, while after 48 h the reduction was not achieved. In this case, there was probably also an increase in the accompanying bacteria for which the phage preparation was not sufficiently specific. After 24 h, there was a decrease in TNB in the control samples, which may have been due to a decrease in water activity because of evaporation. Other reasons for the ineffectiveness of our phage cocktail in reducing bacteria in both washed and unwashed spinach could be the acquisition of resistance by bacterial hosts or the lysogenization of phages in biopreparation. From the side of the composition of the biopreparation, interspecies competition between phages could have occurred [80,81,82].

Research centers around the world are conducting biocontrol tests of food and surfaces for direct contact with the product using broad-spectrum phage cocktails. The research is mainly aimed at the effective elimination of virulent bacterial strains and consists of deliberate contamination of samples with a specific bacterial host strain, against which specific phages are then used. In this way, a reduction of bacteria by two or more log units is achieved. In a study conducted by Gouvea et al. [83], an absorbent food insert containing a mixture of six bacteriophages was used in chilled meat. The system was evaluated for its ability, in vitro, to reduce the initial count of *Salmonella* Typhimurium present in the environment. It was found that the higher the concentration of bacteriophages, the better their effect on the host. A high reduction of *Salmonella* rods after the use of a cocktail of three lytic phages was also demonstrated by Sprincigo et al. [84]. The effectiveness of the phage cocktail in a food matrix either in vitro or in vivo against biofilm was confirmed in the study by Islam et al. [85]. Mangieri et al. [86] evaluated the effectiveness of a phage cocktail in fresh cucumbers. The results showed a reduction of pathogenic Shiga-toxin producing *Escherichia coli* by 1.97–2.01 log CFU g^−1^ at 25 °C and by 1.16–2.01 log CFU g^−1^ at 4 °C within 24 h, suggesting a possibility of using it in the biocontrol of various STEC serogroups. Subsequent studies [87] described the use of broad-spectrum bacteriophage cocktails to control *Campylobacter* sp. in broiler chickens under commercial conditions. In one of the farms included in the study, phage treatment significantly reduced *Campylobacter* levels in the farm cecum in the range of 1–3 log CFU g^−1^ compared to controls. *Campylobacter* isolates from the tested farms remained sensitive to the phages used.

In the production technology of minimally processed plant-based food products, contamination with saprophytic bacteria from raw materials pose a significant problem. These bacteria are not directly dangerous for consumers, but due to their growth and development in the food matrix, they shorten the shelf life, which generates significant financial losses for producers. In the RTE food preservation technology, the manufacturer will not deliberately contaminate food or check which bacterial microflora dominates in each production batch. The phage cocktail used must be so “universal”, i.e., it must contain a wide range of phages selected to be effective against many strains, species, and types of bacteria, both pathogenic and saprophytic, commonly found in products of this type. It should guarantee both the safety and extended shelf life of minimally processed food products.

Currently, there is a lack of scientific publications on the application of phage cocktails to RTE food products to combat saprophytic bacteria. In our previous studies [22], we used a cocktail of twenty-nine different bacteriophages and evaluated its effectiveness in three different food matrices (i.e., broccoli sprouts, spinach leaves, and freshly squeezed carrot-celery juice). In each of the products after 24 h, the phage cocktail significantly reduced TNB. In the experiment of Gientka et al. [21], as in our article, various methods of phage suspension application were used. The spraying method was significantly more efficient on sprouts than the phage-impregnated absorption pad, and the maximum reduction obtained after 48 h of incubation amounted to 1.5 log CFU g^−1^. The use of phage lysate-soaked patches reduced the TNB to approximately 0.27–0.79 log CFU g^−1^. The absorption pad did not show better phage distribution during storage. Perhaps it was the structure of the insert that hindered the release of phages into the product matrix.

Food biocontrol trials carried out on a laboratory scale on the example of selected lettuce mixes suggest that the phage cocktail method may bring the expected reduction in the degree of contamination with accompanying bacteria. In the further stages of research, attention should be paid to the range of bacterial hosts isolated in the course of phage experiments, and those with the broadest spectrum of activity should be selected. To prevent hosts from activating resistance systems to phage infection, phages capable of only lytic development would be desirable. One should also not forget about some dangers related to the use of phages and choose those that do not contain in their genome and will not be able to collect genes responsible for the virulence of bacterial strains from the environment.

## 4. Conclusions

Obtained results indicate that minimally processed plant-based food can be strongly contaminated with saprophytic bacteria, the number of which, even during refrigerated storage, can increase by 2–3 log units, which leads to a reduction of the product’s quality. Among the isolated bacteria, 18.6% were MDR, which indicates the need to monitor the transfer of resistance in the food chain. In addition, the analyzed lettuce mixes were free of pathogenic bacteria for humans. Municipal sewage represents a rich source of phages against various types of saprophytic bacteria commonly found in food and purified viral preparations with a high concentration of phage particles may be used in the future for RTE food preservation. The application of a phage cocktail effectively reduced the growth of bacteria in the three tested products during storage. The ineffectiveness of the phage cocktail in fixing washed and unwashed spinach may indicate the insufficient biodiversity of the phages in the examined preparation. The food products might contain a completely different panel of bacteria than the one that had been isolated in earlier stages of the study. Finally, the bacterial population could have become rapidly resistant or lysogenized by the phages in the biopreparation. Due to the complexity (in terms of the number of bacteriophages) of the phage cocktail, instead of synergy in the bactericidal effect of bacteriophages, interspecies competition of phages could have occurred. Therefore, all these interactions can be significant factors to consider when designing phage cocktails. Subsequent studies should focus on the determination of the range of bacterial hosts for isolated phages and the selection of phages with the widest spectrum of activity. Before phage cocktail can be introduced into standard food production, it is important to select strictly lytic phages that do not encode bacterial endotoxins and virulence genes in their genome. Therefore, a useful technique for this seems to be the whole genome sequencing of phages that should be included in commercial phage biopreparations.

## Figures and Tables

**Table 1:** Microbiological quality of the tested minimally processed plant-based food products during refrigerated storage.

Microbiological Quality of Products x ± SD [log CFU g^−1^]										
Microbiological Analysis	RUC		MLSC		MLSB		WSP		UWSP	
	BED	AED	BED	AED	BED	AED	BED	AED	BED	AED
Total Number of Psychrophilic and/or Psychrotrophic Microorganisms	7.14 ± 0.37 ^b^	9.45 ± 0.45 ^a^	7.35 ± 0.39 ^b^	8.54 ± 0.27 ^a^	7.34 ± 0.05 ^b^	8.52 ± 0.11 ^a^	7.61 ± 0.52 ^b^	9.06 ± 0.40 ^a^	6.29 ± 0.05 ^b^	8.05 ± 0.11 ^a^
Total Number of Mesophilic Microorganisms	7.59 ± 0.59 ^b^	9.84 ± 0.88 ^a^	7.38 ± 0.42 ^b^	8.58 ± 0.24 ^a^	7.29 ± 0.10 ^b^	8.58 ± 0.01 ^a^	7.56 ± 0.53 ^b^	9.07 ± 0.42 ^a^	10.10 ± 0.04 ^b^	10.41 ± 0.06 ^a^
Number of Acidifying Bacteria	5.42 ± 0.61 ^b^	7.09 ± 0.02 ^a^	6.20 ± 1.21 ^a^	7.70 ± 0.36 ^a^	5.17 ± 0.89 ^b^	7.56 ± 0.35 ^a^	5.44 ± 0.23 ^a^	6.42 ± 0.59 ^a^	5.98 ± 0.15 ^b^	9.84 ± 0.05 ^a^
Number of Bacteria from the *coli* Group	5.57 ± 0.37 ^b^	6.91 ± 0.31 ^a^	6.18 ± 0.57 ^a^	7.19 ± 0.35 ^a^	5.75 ± 0.10 ^b^	7.34 ± 0.18 ^a^	5.98 ± 0.51 ^a^	6.65 ± 0.54 ^a^	4.20 ± 0.19 ^b^	6.22 ± 0.07 ^a^
Number of Bacteria from the *Enterobacteriaceae* Family	5.98 ± 0.20 ^b^	6.96 ± 0.37 ^a^	6.26 ± 0.51 ^b^	7.28 ± 0.34 ^a^	5.94 ± 0.17 ^b^	7.56 ± 0.11 ^a^	6.31 ± 0.08 ^a^	6.97 ± 0.70 ^a^	5.32 ± 0.09 ^b^	5.98 ± 0.06 ^a^

Lowercase letters indicate significant differences at *p* < 0.05 (*n* = 3). Abbreviations: RUC—rucola; MLSC—mixed leaf salad with carrot; MLSB—mixed leaf salad with beetroot; WSP—washed spinach; UWSP—unwashed spinach; BED—before the expiration date; AED—after the expiration date.

**Table 2:** Source of isolation and taxonomic identification of bacterial strains.

Bacterial Strain Code	Bacterial Strain Number	Source of Isolation	Bacteria Identification Acc. to MALDI–TOF MS	Bacteria Identification Acc. to *16S* rRNA Sequencing	GenBank Accession Number
RUC-08	KKP 3706	RUC	*Enterobacter* sp.	*Enterobacter cloacae*	OM278533
RUC-09	KKP 3655	RUC	*Citrobacter freundii*	*Citrobacter freundii*	MZ827001
RUC-10	KKP 3800	RUC	*Escherichia coli*	*Escherichia coli*	OM250392
RUC-11	KKP 3801	RUC	*Escherichia coli*	*Escherichia coli*	OM250391
RUC-16	KKP 3825	RUC	*Escherichia coli*	*Escherichia coli*	ON303626
RUC-17	KKP 3651	RUC	*Pantoea agglomerans*	*Pantoea agglomerans*	OP978292
MLSC-01	KKP 3889	MLSC	*Serratia liquefaciens*	*Serratia liquefaciens*	OP999699
MLSC-02	KKP 575	MLSC	*Enterobacter* sp.	*Enterobacter cloacae*	OP935700
MLSC-10	KKP 1384	MLSC	*Rahnella aquatilis*	*Rahnella aquatilis*	OP935751
MLSC-11	KKP 3082	MLSC	*Enterobacter* sp.	*Enterobacter cloacae*	MZ827006
MLSC-13	KKP 3687	MLSC	*Serratia marcescens*	*Serratia marcescens*	OK103977
MLSC-16	KKP 3824	MLSC	*Escherichia coli*	*Escherichia coli*	ON303636
MLSC-17	KKP 591	MLSC	*Serratia liquefaciens*	*Serratia liquefaciens*	OP935744
MLSC-21	KKP 3083	MLSC	*Klebsiella oxytoca*	*Enterobacter ludwigii*	MZ827002
MLSC-22	KKP 590	MLSC	*Pantoea agglomerans*	*Pantoea agglomerans*	OP935743
MLSB-04	KKP 3084	MLSB	*Serratia fonticola*	*Serratia fonticola*	MZ827668
MLSB-07	KKP 589	MLSB	*Pantoea agglomerans*	*Pantoea agglomerans*	OP935741
MLSB-09	KKP 1383	MLSB	*Rahnella aquatilis*	*Rahnella aquatilis*	OP935750
MLSB-10	KKP 3654	MLSB	*Serratia liquefaciens*	*Serratia liquefaciens*	OP978313
MLSB-12	KKP 357	MLSB	*Serratia marcescens*	*Serratia marcescens*	OP935680
MLSB-14	KKP 3688	MLSB	*Escherichia coli*	*Escherichia coli*	OM281784
MLSB-16	KKP 3705	MLSB	*Escherichia coli*	*Escherichia coli*	OM212647
MLSB-18	KKP 581	MLSB	*Citrobacter* sp.	*Citrobacter* sp.	OP935699
MLSB-19	KKP 585	MLSB	*Serratia marcescens*	*Serratia marcescens*	OP935711
MLSB-21	KKP 3650	MLSB	*Escherichia coli*	*Escherichia coli*	OM287487
MLSB-22	KKP 3689	MLSB	*Raoultella terrigena*	*Raoultella terrigena*	OK085529
MLSB-23	KKP 584	MLSB	*Pantoea agglomerans*	*Pantoea agglomerans*	OP935688
MLSB-25	KKP 3684	MLSB	*Enterobacter* sp.	*Enterobacter cloacae*	OM281790
WSP-05	KKP 3685	WSP	*Serratia fonticola*	*Serratia fonticola*	OM281802
WSP-06	KKP 3652	WSP	*Serratia fonticola*	*Serratia fonticola*	OM287486
WSP-07	KKP 3686	WSP	*Enterobacter* sp.	*Enterobacter cloacae*	OM281778
WSP-09	KKP 3802	WSP	*Escherichia coli*	*Escherichia coli*	OM250393
WSP-19	KKP 3707	WSP	*Escherichia coli*	*Escherichia coli*	OM281777
WSP-25	KKP 3691	WSP	*Escherichia coli*	*Escherichia coli*	OM281773
UWSP-07	KKP 3692	UWSP	*Enterobacter cloacae*	*Enterobacter cloacae*	OM281803
UWSP-08	KKP 3887	UWSP	*Pantoea agglomerans*	*Pantoea agglomerans*	OP999723
UWSP-20	KKP 3892	UWSP	*Enterobacter* sp.	*Enterobacter* sp.	OP999695
UWSP-23	KKP 3656	UWSP	*Enterobacter cloacae*	*Enterobacter cloacae*	OM304355
UWSP-30	KKP 3888	UWSP	*Pantoea agglomerans*	*Pantoea agglomerans*	OQ001073
UWSP-33	KKP 3891	UWSP	*Enterobacter* sp.	*Enterobacter* sp.	OP999696
UWSP-36	KKP 3890	UWSP	*Serratia liquefaciens*	*Serratia liquefaciens*	OP999702
UWSP-37	KKP 671	UWSP	*Serratia marcescens*	*Serratia marcescens*	OP935745
UWSP-40	KKP 1218	UWSP	*Rahnella aquatilis*	*Rahnella aquatilis*	OP935749

Abbreviations: RUC—rucola; MLSC—mixed leaf salad with carrot; MLSB—mixed leaf salad with beetroot; WSP—washed spinach; UWSP—unwashed spinach.

**Table 3:** Phenotype resistance of bacterial strains.

Bacterial Strain Number	Source of Isolation	Antibiotic Resistance Pattern	MAR Index	MDR
KKP 3706	RUC	AMP-SAM-AMC	0.11	
KKP 3655	RUC	AMC-MXF	0.07	
KKP 3800	RUC	no resistance *	-	
KKP 3801	RUC	no resistance *	-	
KKP 3825	RUC	no resistance *	-	
KKP 3651	RUC	no resistance *	-	
KKP 3889	MLSC	AMC	-	
KKP 575	MLSC	AMP-SAM-AMC	0.11	
KKP 1384	MLSC	AMP-CPT-CRO-PEF-MXF-AK	0.21	+
KKP 3082	MLSC	AMP-AMC	0.07	
KKP 3687	MLSC	CPT-MXF	0.07	
KKP 3824	MLSC	no resistance *	-	
KKP 591	MLSC	AMP-CPT	0.07	
KKP 3083	MLSC	AMP-CPT	0.07	
KKP 590	MLSC	AMP-AMC-FEP-CPT-CAZ-CT-PEF-MXF-AK	0.32	+
KKP 3084	MLSB	AMP-CPT	0.07	
KKP 589	MLSB	no resistance *	-	
KKP 1383	MLSB	AMP-CPT-CT-CRO-CIP-PEF-MXF-NOR-AK-TOB	0.36	+
KKP 3654	MLSB	no resistance *	-	
KKP 357	MLSB	AMC-CPT-CT	0.11	
KKP 3688	MLSB	no resistance *	-	
KKP 3705	MLSB	no resistance *	-	
KKP 581	MLSB	AMC-MXF-AK	0.11	+
KKP 585	MLSB	AMP-AMC-CPT-CT-PEF-MXF-AK-CN-TOB	0.32	+
KKP 3650	MLSB	no resistance *	-	
KKP 3689	MLSB	AMP-NOR	0.07	
KKP 584	MLSB	no resistance *	-	
KKP 3684	MLSB	AMP-AMC	0.07	
KKP 3685	WSP	AMP-CPT	0.07	
KKP 3652	WSP	no resistance *	-	
KKP 3686	WSP	AMP-SAM-AMC	0.11	
KKP 3802	WSP	no resistance *	-	
KKP 3707	WSP	no resistance *	-	
KKP 3691	WSP	no resistance *	-	
KKP 3692	UWSP	AMP-SAM-AMC-CPT-CT-OFX	0.21	+
KKP 3887	UWSP	AMP	-	
KKP 3892	UWSP	AMP-SAM-AMC	0.11	
KKP 3656	UWSP	AMP-SAM-AMC-CPT	0.14	
KKP 3888	UWSP	no resistance *	-	
KKP 3891	UWSP	CPT	-	
KKP 3890	UWSP	no resistance *	-	
KKP 671	UWSP	AMP-AMC-CPT-MXF-CN-TOB	0.21	+
KKP 1218	UWSP	AMP-CPT-MXF-OFX	0.14	+

* means: no resistance to the tested antibiotics; Notes: AMP–ampicillin; SAM–sulbactam/ampicillin; AMC–amoxicillin/clavulanic acid; FEP–cefepime; CPT–ceftaroline; CAZ–ceftazidime; CT–ceftolozane/tazobactam; CRO–ceftriaxone; CIP–ciprofloxacin; PEF–pefloxacin; MXF–moxifloxacin; OFX–ofloxacin; NOR–norfloxacin; AK–amikacin; CN–gentamycin; TOB–tobramycin; Abbreviations: MAR–Multiple Antibiotic Resistance; MDR–Multi–Drug Resistant strain; RUC—rucola; MLSC—mixed leaf salad with carrot; MLSB—mixed leaf salad with beetroot; WSP—washed spinach; UWSP—unwashed spinach.

**Table 4:** Prevalence of phenotypic antibiotic resistance in bacterial strains.

Antimicrobial Class (*n* = 8)		Antimicrobial Agent (*n* = 28)	Number of Resistant Strains (*n* = 43)	Percentage of Resistant Strains (%)
β–lactam Antibiotics	Penicillins	ampicillin	20	46.5
		sulbactam/ampicillin	6	14.0
		amoxicillin/clavulanic acid	15	34.9
		piperacillin	0	0.0
		piperacillin/tazobactam	0	0.0
		ticarcillin/clavulanic acid	0	0.0
	Cephalosporins	cefepime	1	2.3
		cefotaxime	0	0.0
		ceftaroline	15	34.9
		ceftazidime	1	2.3
		ceftazidime/avibactam	0	0.0
		ceftolozane/tazobactam	5	11.6
		ceftriaxone	2	4.7
	Carbapenems	ertapenem	0	0.0
		imipenem	0	0.0
		meropenem	0	0.0
	Monobactams	aztreonam	0	0.0
Fluoroquinolones		ciprofloxacin	1	2.3
		pefloxacin	4	9.3
		levofloxacin	0	0.0
		moxifloxacin	9	20.9
		ofloxacin	2	4.7
		norfloxacin	2	4.7
Aminoglycosides		amikacin	5	11.6
		gentamycin	2	4.7
		tobramycin	3	7.0
Phenicols		chloramphenicol	0	0.0
Sulfonamides		sulphamethoxazole/trimethoprim	0	0.0

**Table 5:** Isolated phage titers and values of specific growth rate coefficients of the tested bacteria cultures after the addition of specific phages (*n* = 10).

Bacterial Strain Number	Source of Isolation	Phage Titer [PFU mL^−1^]	Appearance of Phage Plaques	Control Culture		Phage–Infected Bacterial Culture			
						MOI 1.0		MOI 0.1	
				∆OD	μ [h^−1^]	∆OD	μ [h^−1^]	∆OD	μ [h^−1^]
KKP 3706	RUC	1.4 × 10^9^	CP	0.397	0.062	0.207	0.033	0.310	0.049
KKP 3655	RUC	6.2 × 10^9^	CP	0.255	0.031	0.136	0.017	0.273	0.025
KKP 3800	RUC	1.1 × 10^7^	CP	0.206	0.021	0.089	0.008	0.105	0.011
KKP 3801	RUC	5.8 × 10^7^	CP	0.300	0.042	0.156	0.021	0.185	0.026
KKP 3825	RUC	1.9 × 10^7^	CP	0.206	0.022	0.019	0.002	0.090	0.008
KKP 3651	RUC	9.4 × 10^7^	CP	0.217	0.019	0.024	0.002	0.094	0.009
KKP 3889	MLSC	6.4 × 10^6^	CP	0.218	0.021	0.150	0.013	0.167	0.015
KKP 575	MLSC	2.2 × 10^8^	CP	0.247	0.020	0.008	0.002	0.168	0.015
KKP 1384	MLSC	3.5 × 10^7^	CP	0.215	0.019	0.152	0.013	0.184	0.015
KKP 3082	MLSC	1.4 × 10^10^	CPH	0.312	0.026	0.147	0.019	0.261	0.023
KKP 3687	MLSC	2.6 × 10^7^	CPH	0.413	0.036	0.287	0.024	0.412	0.032
KKP 3824	MLSC	1.0 × 10^10^	CP	0.258	0.022	0.081	0.009	0.069	0.008
KKP 591	MLSC	6.2 × 10^6^	TP	0.598	0.047	0.486	0.037	0.510	0.041
KKP 3083	MLSC	7.2 × 10^8^	CPH	0.486	0.035	0.160	0.014	0.206	0.022
KKP 590	MLSC	6.4 × 10^6^	CP	0.252	0.022	0.039	0.005	0.158	0.014
KKP 3084	MLSB	4.4 × 10^8^	CPH	0.464	0.039	0.102	0.008	0.173	0.019
KKP 589	MLSB	1.6 × 10^8^	CP	0.142	0.012	0.022	0.004	0.064	0.007
KKP 1383	MLSB	1.2 × 10^9^	CP	0.342	0.029	0.006	0.003	0.242	0.022
KKP 3654	MLSB	8.2 × 10^9^	CPH	0.411	0.032	0.149	0.015	0.355	0.029
KKP 357	MLSB	1.0 × 10^6^	CP	0.089	0.009	0.062	0.007	0.087	0.008
KKP 3688	MLSB	9.0 × 10^9^	CP	0.107	0.011	0.079	0.008	0.024	0.004
KKP 3705	MLSB	1.1 × 10^10^	TP	0.135	0.013	0.053	0.007	0.046	0.006
KKP 581	MLSB	1.3 × 10^9^	CP	0.120	0.011	0.151	0.015	0.059	0.007
KKP 585	MLSB	3.5 × 10^9^	TP	0.152	0.013	0.087	0.008	0.135	0.012
KKP 3650	MLSB	4.6 × 10^7^	CP	0.312	0.027	0.187	0.017	0.201	0.019
KKP 3689	MLSB	4.0 × 10^7^	CPH	0.239	0.022	0.123	0.012	0.044	0.005
KKP 584	MLSB	2.2 × 10^9^	CP	0.215	0.019	0.009	0.002	0.025	0.004
KKP 3684	MLSB	7.6 × 10^5^	CPH	0.247	0.021	0.057	0.007	0.124	0.013
KKP 3685	WSP	2.5 × 10^8^	CP	0.278	0.023	0.169	0.017	0.278	0.023
KKP 3652	WSP	1.7 × 10^8^	TP	0.274	0.024	0.057	0.006	0.191	0.017
KKP 3686	WSP	8.2 × 10^5^	CP	0.115	0.008	0.088	0.011	0.055	0.014
KKP 3802	WSP	5.6 × 10^8^	CP	0.262	0.023	0.161	0.006	0.235	0.006
KKP 3707	WSP	7.4 × 10^7^	CP	0.223	0.019	0.116	0.008	0.221	0.003
KKP 3691	WSP	1.1 × 10^8^	CP	0.317	0.025	0.144	0.011	0.203	0.018
KKP 3692	UWSP	2.0 × 10^9^	CP	0.157	0.013	0.010	0.003	0.094	0.008
KKP 3887	UWSP	1.3 × 10^9^	CP	0.314	0.025	0.017	0.002	0.209	0.019
KKP 3892	UWSP	1.7 × 10^8^	CP	0.436	0.037	0.032	0.003	0.091	0.011
KKP 3656	UWSP	2.2 × 10^9^	CPH	0.318	0.023	0.195	0.008	0.179	0.013
KKP 3888	UWSP	1.1 × 10^10^	CP	0.295	0.026	0.149	0.006	0.112	0.009
KKP 3891	UWSP	5.2 × 10^9^	TP	0.229	0.019	0.166	0.006	0.164	0.009
KKP 3890	UWSP	7.4 × 10^9^	CP	0.233	0.019	0.127	0.002	0.147	0.005
KKP 671	UWSP	8.0 × 10^8^	CP	0.332	0.029	0.164	0.015	0.250	0.022
KKP 1218	UWSP	5.7 × 10^7^	CP	0.238	0.019	0.135	0.015	0.218	0.018

Abbreviations: RUC—rucola; MLSC—mixed leaf salad with carrot; MLSB—mixed leaf salad with beetroot; WSP—washed spinach; UWSP—unwashed spinach; CPH—clear plaques with halo zone; CP—clear plaques; TP—turbid plaques.

**Table 6:** The reduction level of the total number of bacteria (TNB) during storage after phage cocktail application.

RTE Food Products	Time [h]	Total Number of Bacteria x ± SD [log CFU g^−1^]		
		Control	Spraying	Absorption Pad
Rucola	0	8.47 ± 0.11 ^de^	8.35 ± 0.16 ^def^	7.73 ± 0.49 ^f^
	6	9.35 ± 0.23 ^bc^	7.69 ± 0.45 ^f^	8.17 ± 0.17 ^ef^
	24	9.86 ± 0.04 ^b^	8.91 ± 0.06 ^cd^	9.66 ± 0.16 ^b^
	48	11.49 ± 0.13 ^a^	9.46 ± 0.06 ^bc^	9.52 ± 0.08 ^bc^
Mixed Leaf Salad with Carrot	0	8.00 ± 0.03 ^fg^	7.80 ± 0.10 ^g^	8.52 ± 0.18 ^de^
	6	8.48 ± 0.11 ^def^	8.00 ± 0.20 ^fg^	8.15 ± 0.15 ^efg^
	24	8.75 ± 0.25 ^cd^	9.05 ± 0.16 ^bc^	9.30 ± 0.10 ^b^
	48	11.71 ± 0.06 ^a^	9.07 ± 0.34 ^bc^	8.67 ± 0.14 ^cd^
Mixed Leaf Salad with Beetroot	0	8.49 ± 0.13 ^cde^	8.35 ± 0.54 ^de^	8.15 ± 0.09 ^e^
	6	8.51 ± 0.15 ^cde^	7.90 ± 0.04 ^e^	8.25 ± 0.12 ^de^
	24	9.15 ± 0.32 ^bcd^	8.55 ± 0.23 ^bcde^	8.73 ± 0.55 ^bcde^
	48	11.78 ± 0.01 ^a^	9.33 ± 0.56 ^bc^	9.44 ± 0.07 ^b^
Washed Spinach	0	7.73 ± 0.14 ^cd^	6.85 ± 0.02 ^f^	7.24 ± 0.04 ^def^
	6	7.57 ± 0.11 ^de^	6.95 ± 0.22 ^f^	7.13 ± 0.22 ^ef^
	24	8.61 ± 0.27 ^ab^	8.20 ± 0.14 ^bc^	8.33 ± 0.15 ^b^
	48	8.63 ± 0.16 ^ab^	8.87 ± 0.23 ^a^	8.69 ± 0.11 ^ab^
Unwashed Spinach	0	6.98 ± 0.10 ^d^	6.95 ± 0.01 ^d^	6.75 ± 0.12 ^d^
	6	8.26 ± 0.20 ^ab^	6.25 ± 0.44 ^e^	7.65 ± 0.01 ^c^
	24	8.63 ± 0.12 ^a^	8.18 ± 0.13 ^ab^	8.05 ± 0.06 ^bc^
	48	7.86 ± 0.05 ^bc^	8.35 ± 0.19 ^ab^	8.63 ± 0.03 ^a^

Lowercase letters indicate significant differences at *p* < 0.05 (*n* = 3).

## Data Availability

Not applicable.
